# Rationale for Combing Stereotactic Body Radiation Therapy with Immune Checkpoint Inhibitors in Medically Inoperable Early-Stage Non-Small Cell Lung Cancer

**DOI:** 10.3390/cancers14133144

**Published:** 2022-06-27

**Authors:** Alexander Chi, Nam P. Nguyen

**Affiliations:** 1 Department of Radiation Oncology, Capital Medical University’s Affiliated Beijing Chest Hospital, Beijing 101125, China; 2 School of Basic Medical Sciences, Capital Medical University, Beijing 101125, China; 3 Department of Radiation Oncology, Howard University, Washington, DC 20059, USA; nam.nguyen@howard.edu

**Keywords:** early-stage NSCLC, stereotactic body radiation therapy, SBRT, immune checkpoint inhibitors

## Abstract

**Simple Summary:**

The rate of recurrence remains high for lymph node negative early-stage non-small cell lung cancer that are over 2–3 cm in size following stereotactic body radiation therapy (SBRT). This is due to the increased incidence of out-of-field failures, which warrants the addition of systemic therapy. Immune checkpoint inhibitors (ICIs), a class of immunotherapy, may induce a strong distant therapeutic effect known as the “abscopal” effect. This makes them a very suitable class of drugs to be combined with SBRT when treating early lung cancer with high-risk features, such as larger tumor size. In this review, we discuss the rationale and evidence for doing so.

**Abstract:**

Stereotactic body radiation therapy (SBRT) has been widely adopted as an alternative to lobar resection in medically inoperable patients with lymph-node negative (N0) early-stage (ES) non-small cell lung cancer (NSCLC). Excellent in-field local control has been consistently achieved with SBRT in ES NSCLC ≤ 3 cm in size. However, the out-of-field control following SBRT remains suboptimal. The rate of recurrence, especially distant recurrence remains high for larger tumors. Additional systemic therapy is warranted in N0 ES NSCLC that is larger in size. Radiation has been shown to have immunomodulatory effects on cancer, which is most prominent with higher fractional doses. Strong synergistic effects are observed when immune checkpoint inhibitors (ICIs) are combined with radiation doses in SBRT’s dose range. Unlike chemotherapy, ICIs can potentiate a strong systemic response outside of the irradiated field when combined with SBRT. Together with their less toxic nature, ICIs represent a very suitable class of systemic agents to be combined with SBRT when treating ES NSCLC with high-risk features, such as larger tumor size. In this review, we describe the rationale and emerging evidence, as well as ongoing investigations in this area.

## 1. Introduction

Stereotactic body radiation therapy (SBRT), which is also known as stereotactic ablative radiotherapy (SABR), is the standard local treatment of choice for medically inoperable patients with lymph-node negative (N0) early-stage (ES) non-small cell lung cancer (NSCLC). Under image guidance and tumor motion management, SBRT delivers an ablative dose of radiation to the tumor target with a sharp dose gradient at its edge [1]. Therefore, maximizing the probability of local tumor control without causing significant normal tissue damage. Overall, excellent local control has been observed following SBRT delivering an adequate biologically effective dose (BED) in N0 ES NSCLC [2,3]. However, higher incidence of local and distant recurrences has been consistently observed in patients with larger tumors even when a high dose of radiation was delivered to the tumor locally [4,5,6,7,8]. Increased recurrence appears to impact the survival of these patients negatively. Therefore, treatment strategies combining SBRT with additional systemic therapy in medically inoperable N0 ES NSCLC patients with high-risk features, such as larger tumor size, opt to be further explored. In this review, we discuss the rationale and clinical evidence for combining SBRT with a class of immunomodulators, the immune checkpoint inhibitors, in such patients. 

## 2. Clinical Challenges Associated with SBRT for N0 Medically Inoperable ES NSCLC 

Early clinical experience in SBRT has demonstrated worse local control in larger tumors, which appear to require a higher radiation dose [1]. In a study by Andratschke et al., which included 31 patients with cT1 and 61 patients with cT2 tumors, all 10 local recurrences were found in cT2 patients after a median follow up of 21 months, which translated into a 5-year local control of 77% in these patients with T2 lesions [5]. Similarly, all local failures after SBRT were found in cT2 patients in a Nordic phase II study with a longer median follow up period of 35 months [6]. In this study, the 3-year estimate of any failure increased from none in patients with tumors ≤2 cm, to 25% in patients with cT1c tumors, and 40.8% in patients with cT2 tumors. Higher incidence of failures beyond the primary site in larger tumors even when a BED of 112.5 Gy_10_ and 211.2 Gy_10_ were delivered to the tumor’s periphery and isocenter, respectively. Such findings are corroborated in the landmark phase II study, RTOG 0236, which evaluated 55 inoperable patients with peripheral cT1-2, N0, M0 NSCLC who were treated with 54 Gy delivered over 3 daily fractions [7]. In this study, only 1 local failure (cT2 at diagnosis), 3 lobar failures, 2 regional failures, and 11 cases of systemic dissemination were observed after a median follow up of 34.4 months. These translate into the following local control, lobar control, locoregional control, and distant failure estimates at 3 years: 97.6%, 90.6%, 87.2%, and 22.1%, respectively; as well as a 3-year disease-free survival (DFS) of 48.3% and overall survival (OS) of 55.8%. Although the median survival was not reached in cT1 patients, it was only 33.7 months for cT2 patients, which may be associated with the significantly higher rate of distant recurrence in cT2 patients (47.0% vs. 14.7%). The longer-term results of RTOG 0236 reports additional failures after 3 years, which are mainly lobar and regional failures, leading to 5-year estimates of local, lobar, regional, local-regional, and distant recurrence to be 7.3%, 20%, 10.9%, 25.5%, and 23.6%; and 5-year DFS and OS of only 25.5% and 40.0% [8]. At the same time, the incidence of distant recurrence remained as significantly higher in cT2 patients (45% vs. 18.2%). The clinical outcomes following SBRT in selected prospective trials are summarized in Table 1.

There may be several underlying reasons for the higher incidence of failures, and especially distant failures after SBRT in NSCLC patients with larger tumors. First, this may be due to tumor cells and clonogens that remain viable at the primary tumor site for an extended period of time after SBRT, which subsequently disseminates beyond the primary tumor, to regional lymph nodes and distantly. This pattern of dissemination is suggested in the phase II MISSILE-NSCLC trial, which investigated patients with cT1-2 NSCLC’s pathological response after SBRT delivering a dose of 54–60 Gy over 3–8 daily fractions [9]. In this study, a pathological complete response (pCR) of only 60% was achieved in 35 evaluable patients (mostly with cT1 tumors) who underwent lobar or sublobar resection 10 weeks after completing SBRT. The 2-year estimates of local control and OS of 96% and 75% were observed, which are in line with other prospective studies [10,11]. Unexpectedly, a high incidence of regional recurrence was observed. The 2-year estimates of regional and distant control were only 56% and 72%, which appears to be well correlated with the low pCR rate observed. As the majority of patients in this study have cT1 NSCLC, cT2 patients’ pCR rate may be even lower as larger tumors can have more residual viable tumor cells which take longer to be completely eradicated after SBRT; thus, increasing the chance of local-regional and distant failures. 

Secondly, definitive surgery for ES NSCLC is routinely conducted with regional lymph node sampling and/or dissection [12]. However, the risk for recurrence remains significantly higher in patients with pT1c/T2a tumors after surgery [13]. Therefore, the rate of recurrence can be even higher following SBRT, which is delivered in the absence of any regional lymph node assessment. As shown in a study comparing SBRT and surgery after propensity score matching, increased regional recurrence was observed in patients with a mean tumor size of 1.83 cm after SBRT (12.5% vs. 2.7% at 5 years, *p* = 0.017), which is consistent with the rate of upstaging in patients with similar sized peripheral NSCLC after surgery [14]. As shown in a retrospective study of 58 patients with peripheral cT1a-1bN0M0 (≤2 cm) NSCLC, the incidence of upstaging to N1 and N2 was 12% and 3%, respectively [15]. However, the incidence of occult lymph node metastasis increases with increased tumor size. In an analysis of the National Cancer Database (NCDB) data, the rate of occult lymph node metastasis increased significantly from <10% in tumors ≤2 cm to 12.8%, 18.0%, and 20–25% in cT1-4, N0, M0 NSCLC with tumor sizes of 2.1–3.0, 3.1–4.0, and 4.1–7.0 cm, respectively [16]. In a retrospective study assessing patients who were cT1-2aN0M0 based on PET/CT staging, the incidence of occult lymph node metastasis increased significantly from 5% in tumors ≤2 cm to 15.4% in cT2 patients [17]. Overall, the risk of regional recurrence after SBRT may significantly increase as tumor size increases, leading to increased likelihood of distant dissemination. This risk may be further increased with the feature of central location. In a retrospective study of 284 PET/CT staged cT1-2N0M0 NSCLC patients, occult mediastinal lymph node metastasis is identified in 33.3% of patients with centrally located cT2 solid tumors, which may result in a high rate of regional and distant failures if these patients were to undergo SBRT [18]. 

## 3. The Role of Systemic Therapy in ES NSCLC: Clinical Experience with Chemotherapy

The current evidence demonstrates an elevated risk for failure, especially for distant metastasis, outside of the irradiated primary site after SBRT in larger N0 ES NSCLC (1.6–9.14). This risk may be lowered with the addition of systemic therapy, potentially leading to significant improvements in survival in patients with larger primary tumors >2–3 cm. For operable patients with N0 ES NSCLC, such a benefit from chemotherapy has not been demonstrated in prospective randomized trials (Table 2). 

A 5-year OS benefit of 5.4% has been reported in the LACE meta-analysis of 5 adjuvant chemotherapy trials in operable patients with stage I-III NSCLC [19]. However, this survival benefit from adjuvant chemotherapy is mostly limited to patients with stages II-III NSCLC [19,20]. In operable patients with completely resected stage I-III NSCLC, local and distant failures are significantly reduced with adjuvant chemotherapy, which correlated with significant OS and DFS benefits at 5 years [21,22]. In the International Adjuvant Lung Cancer Trial (IALT), chemotherapy led to a 4.4% and 6.2% reduction in local recurrence and 4.2% and 3.7% reduction in distant metastasis (mainly extra-cranial metastasis) at 5 and 8 years, respectively, which are associated with an absolute gain in 5-year DFS and OS of 4.3% and 3.9%, respectively [11]. However, chemotherapy’s benefits on OS and DFS disappeared, while a detrimental effect on OS surfaced after 5 years in the IALT. Higher percentages of OS and DFS benefits from adjuvant chemotherapy were observed in the Adjuvant Navelbine International Trialist Association (ANITA) trial, which randomized 840 operable stage IB-IIIA NSCLC patients to observation or four cycles of cisplatin plus vinorelbine [22]. The absolute OS benefit was 8.6% and 8.4%, while the absolute DFS benefit was 8.7% and 5.5% at 5 and 7 years in this trial. Local relapse was significantly reduced (12% vs. 18%, *p* = 0.025) along with the incidence of relapse in the lungs (22% vs. 28%, *p* = 0.004) and bone metastasis (4% vs. 11%, *p* = 0.0001) with adjuvant chemotherapy. However, chemotherapy significantly impacted survival only in node-positive patients and those with tumors over 5 cm in size in this trial. 

In a subgroup analysis of the LACE-vinorelbine cohort, N0 tumors between 3 and 5 cm have been associated with a 5-year survival benefit of only 1.8% from adjuvant chemotherapy [23]. The lack of benefit from adjuvant chemotherapy in T2N0 patients was further validated in JBR.10 and CALGB 9633, which enrolled only patients with ES NSCLC [24,25]. In the subset of patients with tumor size of ≥4 cm, adjuvant chemotherapy has been correlated with a 31% reduction in the risk of any recurrence and death upon exploratory analysis in CALGB 9633, which only enrolled N0 patients with a median tumor size of 4 cm [25]. However, this subset of patients had a mean tumor size above 5.5 cm. In addition, CALGB 9633 was not powered to detect any clinically meaningful small differences in OS and DFS. Therefore, adjuvant chemotherapy was only recommended to be considered in the presence of tumor size of >4 cm and other high-risk surgical-pathological features (poor differentiation, vascular invasion, visceral pleural involvement, Nx status, and wedge resection) for N0 ES NSCLC by the NCCN guidelines [26]. In the multicenter randomized Chemotherapy for Early Stages Trial (CHEST), a lack of clinical benefit in ES NSCLC was also found with neo-adjuvant chemotherapy [27]. 

Despite a lack of benefit in prospective trials which employed cisplatin-based regimens, a potential benefit from chemotherapy in patients with >T1bN0M0 NSCLC has been suggested in a more recent large retrospective study from Japan that included 1278 patients [13]. In this study, the 5-year RFS increased from 73.8% to 81.4% (*p* = 0.023) in operable patients with stage I NSCLC per the 8th edition of AJCC staging criteria in the presence of pT1c/T2a or lymphovascular invasion. On the contrary, adjuvant chemotherapy did not lead to any significant increase in 5-year RFS (98.1% vs. 95.7%, *p* = 0.30) in patients with pT1a/b tumors and no lymphovascular invasion. In total, 157 of the 305 patients who received adjuvant chemotherapy received oral tegafur-uracil (UFT) therapy in this study. Thus, suggesting the possibility for more noticeable clinical benefit with less toxic systemic agents in N0 ES NSCLC between 2 and 3 cm in size. This is also corroborated in the LACE meta-analysis, which demonstrated a significant correlation between chemotherapy’s benefit with patients’ performance status [19]. 

Data on the use of adjuvant chemotherapy with SBRT in N0 ES NSCLC has been mostly limited to analyses of the NCDB data. In one such analysis, median survival was significantly prolonged with adjuvant chemotherapy (19 vs. 15.9 months, *p* < 0.001) in patients with tumors ≥ 4 cm [28]. However, chemotherapy led to shorter median survival in patients with tumors < 4 cm (24.3 vs. 28.5 months, *p* < 0.001). Although a survival benefit was not demonstrated, significant reduction in regional and distant failures in patients with >T1N0M0 NSCLC was found in a retrospective study, which supports the hypothesis that the rate of recurrence following SBRT may be reduced with systemic therapy in this patient population [29]. However, the toxicity profile of combining chemotherapy and SBRT is largely unknown, with a general concern for severe toxicity. As shown in the LACE meta-analysis, chemotherapy benefits patients with good performance status only [19]. Unlike operable patients, most patients undergoing SBRT tend to be older and less fit for chemotherapy medically. Overall, clinical evidence does not provide any strong justification for combining SBRT with chemotherapy in patients with N0 ES NSCLC. Less toxic systemic agents that may have a significant out-of-field therapeutic effect when combined with SBRT opt to be further explored. 

## 4. Immunomodulatory Effects of SBRT and Their Augmentation by Immune Checkpoint Inhibitors: Preclinical Evidence

Ablative doses of irradiation have been consistently shown to induce T-cell-mediated antitumor immunity at the site of primary tumor and sites of distant metastasis in vivo [30,31,32]. In a murine model of poorly immunogenic metastatic lung cancer, a single dose of 60 Gy induced both local and distant control when dendritic cell (DC) expansion was simultaneously stimulated, which led to significant improvement in median survival from <49 days with irradiation alone to >136 days [30]. However, this phenomenon was not observed in immune-deficient mice. In murine models, ablative doses of radiation led to not only significant local CD8^+^ T cell infiltration that is accompanied by the reduction in myeloid-derived suppressor cells (MDSCs) and tumor-associated macrophages (TAMs), but also prominent distant tumor eradication in a CD8^+^ T-cell-dependent manner that requires DC-induced T cell priming [31,32]. In vivo, the distant, or “abscopal” effect has been significantly suppressed by chemotherapy [31]. Additionally, tumor regression following irradiation is attenuated with multiple fractions of moderately-low radiation doses (e.g., 5 Gy × 4 fractions). These observations may be related to the detrimental effects on activating T cells and antigen presenting cells (APCs), as well as the induction of suppressive immune cells resulting from chemotherapy and prolonged course of low-dose irradiation [33,34]. In vitro, major histocompatibility complex class I (MHC I) expression and the level of tumor-associated antigens (TAAs) in tumor cells increased after irradiation with 10–25 Gy [35]. Such increase is radiation dose dependent, with shorter times taken for MHC I expression to plateau at higher fractional doses above 20 Gy. Significant reduction in tumor volume was observed only when adoptive transfer of cytotoxic T lymphocytes (CTLs) specific to tumor-associated antigens (TAAs) was combined with high single-dose irradiation delivering 20 Gy. In addition to TAA-induced cross priming and activation of CTLs, high dose irradiation’s ability to induce immunogenic tumor cell death also resulted from the activation of the cGMP-AMP synthase (cGAS)-stimulator of interferon genes (STING) pathway by cytosolic double stranded (ds) DNA from tumor cells post-irradiation, which resulted in increased type I interferon (IFN) expression, recruitment of DCs, and subsequent cross presentation and CD8^+^ T cell activation [36,37,38,39]. However, any abscopal effect may be abrogated with doses above 10 Gy given in a single fraction due to the clearance of cytosolic dsDNA by DNA exonuclease Trex1 that is upregulated by high radiation doses [39]. 

Tumor-specific antigen induced T cell activation is held closely in check by immune checkpoints, a series of co-stimulatory and inhibitory receptor/ligand pairs [40]. Immune checkpoint inhibitors have been used to augment antitumor immune response and overcome immune exhaustion in an overall suppressive tumor immune microenvironment [40,41,42]. The most targeted immune checkpoints are cytotoxic T-lymphocyte-associated antigen-4 (CTLA-4) and programed cell death protein-1 (PD-1). CTLA-4 is upregulated on the T cell surface upon T-cell receptor (TCR) activation with the costimulatory signal resulting from binding of CD28 on T cell surface with B7 ligands B7-1 (CD80) and B7-2 (CD86) on APCs [42]. CTLA-4 then competes with CD 28 for binding of B7 ligands, leading to attenuated T cell activation. Unlike CTLA-4, PD-1 primarily affects T cell activity in its effector phase in peripheral tissue and within the tumor [42,43]. Upon T cell activation, PD-1 is expressed and binds to its ligands PD-L1 and PD-L2 in nonlymphoid tissues. A negative signal is then generated to attenuate T cell activation in peripheral tissues, including the tumor microenvironment [TME]. 

In preclinical studies, immune checkpoint blockade increased the observation of an abscopal effect in addition to added local response when combined with local tumor irradiation (Table 3). Consistently increased local and distant responses with increased frequency of complete tumor regression were demonstrated in murine models of poorly immunogenic breast cancer when multi-fraction RT was combined with an anti-CTLA-4 antibody [44,45]. In one study, significantly reduced lung metastasis was observed when one single dose of 12 Gy was combined with an anti-CTLA-4 antibody given 1–7 days after irradiation [44]. Although the local control was improved by a second dose of 12 Gy delivered after 48 h, a survival benefit was induced only after this RT regimen was combined with an anti-CTLA-4 antibody. In addition, the combined treatment further enhanced the local response with an increased number of mice achieving complete tumor regression. Dose fractionation also affected tumor response when the combined treatment was delivered. When a single dose of 20 Gy was compared with 8 Gy × 3 fractions or 6 Gy × 5 fractions in the setting of combining RT with an anti-CTLA-4 antibody, increased local and distant responses were only observed with multi-fractional regimens [45]. The abscopal effect induced was strongest when 8 Gy × 3 fractions were delivered with concurrent drug treatment, which led to significantly increased tumor infiltration by CD4^+^ and CD8^+^ T cells at distant sites. It was further shown that tumor eradication at both local and distant sites was mediated by tumor antigen-specific CD8^+^ T cells [44,45].

PD-L1 expression by tumor cells at the primary site is induced upon local irradiation [46,47,48]. Upon multi-fractional low-dose irradiation, this response was shown to be mediated by CD8^+^ T cells within the TME through IFN-γ signaling [46]. Significant antitumor activity has been demonstrated when RT was combined with the concurrent administration of anti-PD-1 or anti-PD-L1 antibodies in murine cancer models [46,47,48,49,50]. Comparing to RT or anti-PD-(L)1 antibody alone, increased local tumor control, out-of-field response, and prolonged survival were observed after combined treatment of RT and anti-PD-(L)1. Significant distant response and improvement in survival only occurred when RT was combined with PD-(L)1 blockade. With the combined treatment, increased tumor infiltration by tumor antigen-specific CD8^+^ T cells at both the primary and distant tumor sites was observed [46,47,48]. As shown in vivo, shared T cells clones between primary and distant tumors are increased at the primary site only upon irradiation alone. However, their number also increased at distant tumor sites after combined treatment with RT and an anti-PD-1 antibody [47]. Locally, such combined treatment also led to a significant reduction in MDSCs and regulatory T cells (Tregs) [48,49]. This reduction in suppressive immune cells may be induced by T-cell-derived cytokines, such as TNF [48]. Ablative doses have been shown to increase tumor antigen presentation in a dose-dependent manner. This occurs within the TME and in the draining lymph nodes, resulting in tumor infiltration by CD4^+^ and CD8^+^ T cells [49]. The best local control was observed when ablative doses are combined with PD-1 blockade. This was observed to be related to local Treg eradication in the presence of increased CD8^+^ T cell infiltration, leading to a higher CD8^+^ T cell to Treg ratio within the primary tumor [49]. Higher incidence of the abscopal effect along with improved survival were observed when single high-dose irradiation was combined with PD-1 blockade [48,50]. As observed with lower radiation doses, the abscopal effect was mediated by tumor antigen-specific CD8^+^ cells. Increased PD-1 expression in effector CD8^+^ T cells along with increased PD-L1 expression in tumor cells was observed at both the primary and distant tumor sites after single high-dose irradiation [50]. Such over-expression represents an overall state of immunosuppression mediated by the PD-1–PD-L1 axis upon irradiation. Therefore, targeting the PD-(L)1 immune checkpoint along with irradiation may lead to profound systemic effects, which makes combining anti-PD-(L)1 immune checkpoint inhibitors (ICIs) with SBRT a very rational treatment strategy for N0 ES NSCLC with high-risk features, such as larger tumor size. 

## 5. Clinical Evidence for Combining SBRT with Immune Checkpoint Inhibitors 

Chemotherapy, which sensitizes the tumor within the irradiated volume to radiotherapy, has not been shown to lower the incidence of distant metastases following concurrent chemoradiation in locally advanced NSCLC [51,52,53]. Strategies to combine radiotherapy and ICIs in the treatment of lung cancer have been under intense investigation in recent years [54,55,56]. When combined with radiotherapy, ICIs have been shown to induce a significant and durable distant response in NSCLC patients. The median time to death or distant metastasis (TTDM) was significantly prolonged from 17.7 to 36.5 months when adjuvant Durvalumab, an anti-PD-L1 antibody, was administered after conventional concurrent chemoradiation (cCRT) in patients with locally advanced unresectable NSCLC [57]. This corresponded to a 41% reduction in death or distant metastasis as the incidence of new lesions decreased from 33.3% to 24.2%, and the incidence of brain metastases decreased from 11.8% to 6.5%. Such decrease in distant metastasis directly resulted in significantly improved 5-year progression-free survival (PFS) (33.1% vs. 19.1%) and OS (42.9% vs. 33.4%). The safety of combining an anti-PD-(L)1 antibody with SBRT in the treatment of NSCLC has been demonstrated in several studies [58,59,60,61]. The incidence of severe treatment-related toxicities is approximately 75–80% with cCRT [53]. On the contrary, concurrent anti-PD-(L)1 ICI appears to be associated with much less severe toxicities when combined with SBRT [58,59,60,61]. 

A dramatic increase in the rate of an abscopal response (ARR) was observed in stage IV NSCLC patients after a median follow up of 33 months after combined treatment with Pembrolizumab, anti-PD-1 ICI, and SBRT [62]. With the addition of SBRT, the ARR increased from 19.7% to 41.7% (*p* = 0.0039), while the abscopal control rate (ACR) increased from 43.4% to 65.3% (*p* = 0.0071). This led to increased median PFS from 4.4 to 9.0 months (*p* = 0.045) and median OS from 8.7 to 19.2 months (*p* = 0.0004). Comparing to other radiotherapy regimens, SBRT delivering 12.5 Gy × 4 fractions was prognostic of better PFS upon multivariate analysis. Significant clinical impact on the primary tumor was also observed in operable cT1-2N0M0 NSCLC patients who received concurrent Durvalumab and SBRT delivering 8 Gy × 3 fractions, which correlates with a BED of only 43.2 Gy_10_ [63]. An impressive pCR rate of 50% was observed with this neoadjuvant regimen, which approximates that following SBRT alone delivering much higher BEDs [9]. 

Overall, early clinical findings confirmed ICIs’ ability to potentiate a strong distant tumor response with added local antitumor activity when combined with SBRT, and such effects appear to be maximal when an ICI is delivered concurrently (Table 4). These findings provide early evidence supporting the concept of reducing out-of-field recurrence in high-risk N0 ES NSCLC following SBRT by combining SBRT with an ICI, which opt to be further investigated in prospective trials.

Several clinical trials investigating the feasibility and efficacy of combining SBRT with an ICI in the treatment of patients with N0 ES NSCLC are currently ongoing (Table 5). 

The overall safety of combining an anti-PD-L1 immune checkpoint inhibitor with SBRT concurrently or adjuvantly has been reported in abstract format [63,64]. At present, three randomized trials evaluating the efficacy of adding an ICI to SBRT in N0 ES NSCLC are ongoing. Among them, PACIFIC-4/RTOG 3515 (NCT03833154) and KEYNOTE (KN)-867 (NCT03924869) are placebo-controlled and do not select patients based on high-risk features [65,66,67]. PACIFIC-4 was initially designed to have patients receive adjuvant Durvalumab or placebo for two years after SBRT over 3, 4, 5, or 8 fractions, but was then modified to concurrent administration of Durvalumab or placebo with SBRT for two years [65,66]. A separate EGFR-mutant cohort also exists in PACIFIC-4, which will be assigned to adjuvant Osimertinib for three years after SBRT. KN-867 randomizes ES NSCLC patients to SBRT delivered in 3, 4, 5, or 8 fractions combined with concurrent and adjuvant placebo or Pembrolizumab for up to 1 year [67]. On the contrary, the SWOG/NRG S1914 (NCT04214262) randomizes ES NSCLC patients with tumors ≥ 2 cm, tumor max SUV ≥ 6.2, moderately/poorly differentiated or undifferentiated histology to either SBRT delivered in 3–5 fractions alone or SBRT combined with neoadjuvant, concurrent, and adjuvant atezolizumab for 6 months [68]. As shown in the initial phase I study selecting an appropriate dose of Atezolizumab to be used in this combination regimen, a dose at 1200 mg given every 21 days resulted in one case of dose limiting toxicity (DLT), a grade 3 rash, in 12 patients evaluable for DLT [64]. Thus, providing evidence of feasibility of the SWOG regimen, while the SWOG trial’s results are eagerly awaited. Worth noting, none of these trials select patients based on their PD-L1 status, while only PACIFIC-4 limits ICI to patients without EGFR mutations. Additionally, all of these trials use multiple SBRT dose fractionation regimens based on tumor location. 

## 6. Conclusions

The incidence of recurrence is higher in medically inoperable patients with N0 ES NSCLC that are larger in size following SBRT. The pattern of failure is predominantly distant. The benefit of systemic chemotherapy has been shown to be only marginal in N0 ES NSCLC patients with its use not clearly supported in the medically inoperable population. SBRT may induce immunogenic effects on cancer, which are augmented by immune checkpoint inhibitors, leading to a strong systemic effect, known as the “abscopal effect”. This occurs along with enhanced antitumor activity locally. Such effects are most prominent when an ICI is delivered concurrently with irradiation. Early clinical evidence has been consistent with preclinical findings, which supports combining SBRT with ICIs when treating N0 ES NSCLC with high-risk features, such as larger tumor size. Clinical investigations in this area are currently ongoing. 

## Figures and Tables

**Table 1:** Clinical outcome in selected prospective trials on SBRT for cT1-2N0M0 NSCLC.

Study	N	Dose	Patterns of Failure	Survival
Nordic phase II study [6]	57	45 Gy/3 Frx	3 yr Estimates: Local control: 92%; all failures are T2; Any failure: (T2 vs. T1 *p* = 0.027) T2a = 40.8%; T1b = 25.4%; T1a = 0.0%	3 yr Estimates: PFS: 52% OS: 60% CSS: 88%
RTOG 0236 [7,8]	55	54 Gy/3 Frx	3 yr Estimates: Local control: 97.6%; 1 failure, T2 Lobar control: 90.6% Local-regional control: 87.2% DM: 22.1% (T1: 14.7%; T2: 47%) 5 yr Estimates: Local control: 92.7% Lobar control: 80% Local-regional control: 74.5% DM: 23.6% (T1: 18.2%; T2: 45.5%)	3 yr Estimates: DFS: 48.3% OS: 55.8% 5 yr Estimates: DFS: 25.5% OS: 40.0%
MISSILE-NSCLC trial [9]	35	54 Gy/3 Frx 55 Gy/5 Frx 60 Gy/8 Frx	pCR: 60% 2 yr Estimates (surgery group): Local control: 100% Regional control: 53% DM: 24%	2 yr OS: 77%
RTOG 0915 [10]	39 and 45	34 Gy/1 Frx and 48 Gy/4 Frx	2 yr Estimates: Local control: 97.4% and 97.8%	2 yr Estimates: DFS: 56.4% and 71.1% OS: 61.3% and 77.7%
RTOG 0813 [11]	38 and 33	57.5 Gy/5 Frx and 60 Gy/5 Frx	3 yr Estimates: Lobar control: 86.7% and 84.7%	3 yr Estimates: PFS: 35.7% and 32.5% OS: 51.6% and 54.0%

Frx: fractions; yr: year; PFS: progression-free survival; OS: overall survival; CSS: cancer-specific survival; DFS: disease-free sur vival; DM: distant metastases; pCR: pathological complete response.

**Table 2:** Marginal benefits from adjuvant or neoadjuvant chemotherapy for ES NSCLC in randomized trials.

Study	N	Chemotherapy	Clinical Efficacy
IALT (21)	1867	Adj. CDDP based regimen × 3–4 cycles	Overall: ↑5 yr DFS 4.3%; ↑5 yr OS 3.9%; HR of death: 0.86 (1st 5 yrs); 1.45 (after 5 yrs) DFS HR for cT1-2N0M0 NSCLC: 0.92
ANITA (22)	840	Adj. CDDP/Vinorelbine × 4 cycles	Overall: 5- and 7-year absolute survival benefit: 8.6% and 8.4% 5- and 7-year absolute DFS benefit: 8.7% and 5.5% Chemo. vs. Obs. 5 yr OS for pT2N0M0: 62% vs. 64% 5 yr OS per N status: N0: 58% vs. 61% N1: 52% vs. 36% N2: 40% vs. 19%
JBR. 10 (24)	482	Adj. CDDP/Vinorelbine × 4 cycles	No survival benefit from chemotherapy in N0 tumors >3 –7 cm (HR 1.03).
CALGB 9633 (25)	344	Adj. Carboplatin/Paclitaxel × 4 cycles	Chemo. vs. Obs. (>3–7 cm). 5 yr OS: 60% vs. 58% 5 yr DFS: 52% vs. 48% ≥4 cm group (*p* < 0.05): HR for death: 0.69 HR for DFS: 0.69
CHEST (27)	246	Neoadj. CDDP/Gemcitabine × 3 cycles	No PFS or OS observed in N0 patients with tumors >3–7 cm

CDDP: cisplatin; yr: year; DFS: disease-free survival; HR: hazard ratio; Chem.: chemotherapy; Obs.: observation; OS: overall survival; PFS: progression-free survival.

**Table 3:** Immunogenic effects of RT or RT and immune checkpoint inhibitor combinations observed in preclinical studies.

	Local	Distant	Impact on Survival	Alterations in Local and/or Distant TME
RT-conv. dose [31,32,46,47]	Only delayed tumor growth	↑DM	↓Survival when compared with ablative doses	↓CD8^+^ T cell (acute) and ↑MDSCs locally ↑PD-L1 expression by tumor cells at the primary site mediated by CD8^+^ T cell released IFNγ ↑PD-L1 expression by MDSCs at the primary site.
RT-ablative dose [30,31,32,35,36,37,38,39,48,49]	Durable local control Increased local control with multi-frx regimen	Any distant effects are abrogated by chemotherapy DM significantly decreased when RT is combined with an immunomodulator	↑Survival only in the presence of CD8^+^ T cells; and when RT is combined with activating immunomodulators	↑TAAs and tumor-specific Ag presentation by MHC I within the local TME ↑T cell priming in draining lymph nodes ↑CD4^+^ and CD8^+^ T cell infiltration at the local tumor site Batf3 CD8^+^ DCs and IFNγ are required for any curative effect from RT ↓MDSC and TAMs, but ↑Tregs ↑dsDNA induced IFN β release mediated by cGAS-STING, leading to cross priming ↓cytosolic dsDNA mediated by Trex1 with single dose irradiation >10–12 Gy ↑PD-L1 expression by tumor cells ↑PD-1 expression by CD8^+^ T cells at both primary and distant sites
RT + anti-CTLA-4 [44,45]	Most tumor response and CR. Best response with multi-frx RT	Significant ↓ DM only with the combined Rx Best response with multi-frx RT	Significant improvement in survival is only observed with the combined Rx	Distant effect is CD8^+^ T cell dependent Combined Rx led to ↑ infiltration by CD4^+^ and CD8^+^ T cells distantly Combined Rx led to ↑tumor Ag specific CD8^+^ T cells in lymphoid tissue
RT + anti-PD-(L)1 [46,47,48,49,50]	Best tumor response with further improvement over RT alone	Best distant response with most distant CR	Best survival when compared with either Rx alone Best survival with concurrent Rx	Local and distant effects are CD8^+^ T cell dependent (infiltrating > residing) ↑tumor Ag specific CD8^+^ T cells locally with RT alone but increased further locally with distant ↑ only after combined Rx ↑PD-L1 expression by tumor cells and MDSCs at both the primary and distant sites ↓↓↓MDSCs at the primary site mediated by CD8^+^ T cells ↓Tregs at the primary site

TME: tumor micro-environment; RT: radiotherapy; conv.: conventionally fractionated; DM: distant metastases; TAA: tumor-associated antigens; MDSC: myeloid-derived suppressor cell; Ag: antigen; TAM: tumor-associated macrophage; Treg: regulatory T cells; multi-frx: multi-fraction; Rx: treatment.

**Table 4:** Emerging clinical evidence supporting combining SBRT with ICIs in high-risk N0 ES NSCLC.

Study	N	Stage	Treatment	Response	Toxicity	Survival
Pembro-RT trial (phase II)/ MDACC phase I/II trial [58,59,62]	Pembro: 76 Pembro + SBRT: 72	IV	Pembro vs. SBRT + Adj. Pembro (Pembro-RT)/Concurrent Pembro + SBRT/HypoFrx-RT (MDACC trial)	ARR: 19.7% vs. 41.7% (*p* = 0.0039) ACR: 43.4% vs. 65.3% (*p* = 0.0071)	Grade 3–5 irAEs: Pembro-RT: 17% MDACC trial: 19% after concurrent Pembro-SBRT	Median PFS: 4.4 vs. 9.0 months (*p* = 0.045). Prognostic factor for PFS: SBRT with 50 Gy/4 Frx Median OS: 8.7 vs. 19.2 months (*p* = 0.0004)
Cornell randomized phase II trial [61]	Dur: 30 Dur + SBRT: 30	I-IIIA	Neoadj. Dur × 2 cycles vs. Dur × 2 cycles + SBRT (8 Gy × 3 Frx)	MPR: 6.7% vs. 53.3% (*p* < 0.0001) CR after Dur + SABR: 50%	Grade 3–4 AEs: 17% vs. 20%	

MDACC: MD Anderson Cancer Center; Pembro: Pembrolizumab; Adj.: adjuvant; ARR: abscopal response rate; ACR: abscopal control rate; PFS: progression-free survival; OS: overall survival; Dur: Durvalumab; Neoadj.: neoadjuvant; MPR: major pathological response; pCR: pathological complete response; AE: adverse effect.

**Table 5:** Clinical trials investigating SBRT and ICI combinations for N0 ES NSCLC.

	Phase	Tumor Stage	Study Drug	Drug Schedule and Duration	Primary End Point
NCT02599454 (active, not recruiting)	I	cT1-2N0M0: ≥2 cm, or SUV_max_ ≥ 6.2, or Mod-poorly diff/undifferentiated	Atezolizumab	Neoadj, concurrent, and adj. × 6 cycles combined with SBRT (4–5 frx)	MTD
NCT03050554 (terminated)	I/II	cT1-T2aN0M0	Avelumab	Concurrent and adj. 6 cycles with SBRT (4–5 frx)	Safety and RFS
NCT03148327 (active, not recruiting)	I/II	cT1-3N0M0	Durvalumab	Phase II: SBRT vs. SBRT (3, 4, 10 frx) + neoadj. (5 days before), concurrent, and adj. ICI × 5 cycles	Safety and median PFS
NCT03383302 (was recruiting between 2017–2020)	Ib/II	cT1-3N0M0 (≤5 cm, AJCC 7th ed.)	Nivolumab	Adj. starting within 24 h from last frx of SBRT (3–5 frx) for 12 months	≥grade 3 pneumonitis at 6 months after SBRT
NCT04271384 (recruiting)	II	cT1-2aN0M0 (≤4 cm)	Nivolumab	Concurrent × 3 doses with SBRT (3, 5, or 8 frx) before surgery	pCR rate
NCT03110978 (recruiting)	II	cT1-3N0M0; Isolated recurrence	Nivolumab	Concurrent and adj. × 12 weeks (4 cycles) with SBRT (4 or 10 frx)	EFS
NCT04944173 (not yet recruiting)	II	cT1-2N0M0	Durvalumab	4 cycles of ICI, SBRT (4 frx) concurrent with 2nd cycle	Overall recurrence rate at 18 months
NCT03446547 (recruiting)	II	cT1-2N0M0	Durvalumab	SBRT (3–4 frx) vs. SBRT + adj. ICI × 12 months	TTP
NCT03833154 (recruiting)	III	cT1-3N0M0	Durvalumab	SBRT (3–5, 8 frx) vs. SBRT + adj. ICI × 24 months	PFS
NCT04214262 (recruiting)	III	cT1-T3N0M0	Atezolizumab	SBRT (3–5 frx) vs. SBRT + neoadj., concurrent, and adj. ICI for 8 cycles	OS
NCT03924869 (recruiting)	III	cT1-T3N0M0	Pembrolizumab	SBRT (3–5, 8 frx) vs. SBRT + concurrent and adj. ICI × 12 months	EFS, OS

Mod-poorly diff: moderately to poorly differentiated; Neoadj.: neoadjuvant; Adj.: adjuvant; frx: fraction; MTD: maximum tolerated dose; RFS: recurrence-free survival; PFS: progression-free survival; CR: complete response; EFS: event-free survival; TTP: time to progression; OS: overall survival.
